# Identification of a gene signature for the prediction of recurrence and progression in non-muscle-invasive bladder cancer

**DOI:** 10.1186/s43556-022-00069-0

**Published:** 2022-03-15

**Authors:** Emiliano Dalla, Raffaella Picco, Giacomo Novara, Fabrizio Dal Moro, Claudio Brancolini

**Affiliations:** 1 Department of Medicine, Università degli Studi di Udine, P.le Kolbe 4 –, 33100 Udine, Italy; 2 Department of Surgery, Oncology and Gastroenterology, Urology Clinic, University of Padova, Padova, Italy

Dear Editor,

Non-muscle-invasive bladder cancer (NMIBC) is the most frequently diagnosed bladder cancer and intravesical instillation of Bacillus Calmette-Guérin (BCG) plus transurethral resection (TUR) is the standard therapeutic approach [1]. Since delayed interventions are often associated with lower patient survival, knowing which tumors develop aggressive behavior is essential to better manage high-risk patients. Gene expression, epigenetic and proteomic screenings have been exploited to identify prognostic biomarkers [1, 2]. Unfortunately, few progresses have been made so far. The recent publication of new gene expression data and associated clinical information [2] prompted us to identify a novel gene signature possibly predicting NMIBC recurrence and progression, driving the selection of the most appropriate therapeutic strategy.

In this study, we examined the NMIBC RNAseq dataset GSE154261 [2]. Compared to other studies, cohort’s clinical stage was homogeneous with only high-grade stage T1 (Supplementary Table 1) and had a high percentage (74%) of BCG-treated patients (*n* = 73/99). However, the lack of recurrence and progression data homogeneity forced us to simplify and merge data into a single “recurrence/progression” class. All patients were newly diagnosed (no prior bladder cancer, intravesical chemotherapy, or immunotherapy exposure) and all received at least 6 doses of full-strength BCG. We stratified BCG-treated patients based on the presence (“Yes”, *n* = 31) or absence (“No”, *n* = 42) of cancer recurrence/progression after treatment (i.e. the development of high-grade bladder tumor after the last TUR) (Supplementary Table 2).

We considered the differentially expressed genes (DEGs) and including them in univariate/multivariate regression analysis, subsetting patients based on the received treatments and, finally, stratifying them based on the time of recurrence/progression. However, despite significant survival and regression analyses results, three identified gene signatures could not classify the two groups of patients upon unsupervised hierarchical clustering (Supplementary Figs. 1, 2 and 3).

We then changed our approach, emphasizing the gene expression differences between the two groups of patients. First, we identified genes (*n* = 451) that had a high Spearman correlation (R > 0.5, *p*-value< 0.05), between gene expression and recurrence/progression, in patients who experienced recurrence/progression. Afterwards, we selected those up-regulated (logFC> 1.0, *p*-value< 0.05; *n* = 251) in the “< 1 year recurrence” versus “> 5 years free status” patients. 59 genes satisfied both requirements (Fig. 1a). Finally, we performed a univariate Cox regression analysis with two different significance thresholds, defining the subsets of up-regulated genes with HR > 1.0 (i.e. associated with an increased probability of tumor relapse) finding *n* = 8 (*p*-value< 0.01) and *n* = 28 (*p*-value< 0.05) genes (Fig. 1a). We evaluated the ability of genes identified in each step to properly stratify patients upon unsupervised hierarchical clustering, finally selecting the 28-genes signature as the best performing. Figure 1b shows how this signature distinguishes the two original classes of patients. The classifying potential of this gene list was also used on a larger dataset including all patients with < 1 year recurrence/progression (*n* = 18) and with a > 5 years free status (*n* = 11), regardless of the treatment received. Both the median PI and the optimal cut-point provided significant results (Fig. 1c).

**Fig. 1:**
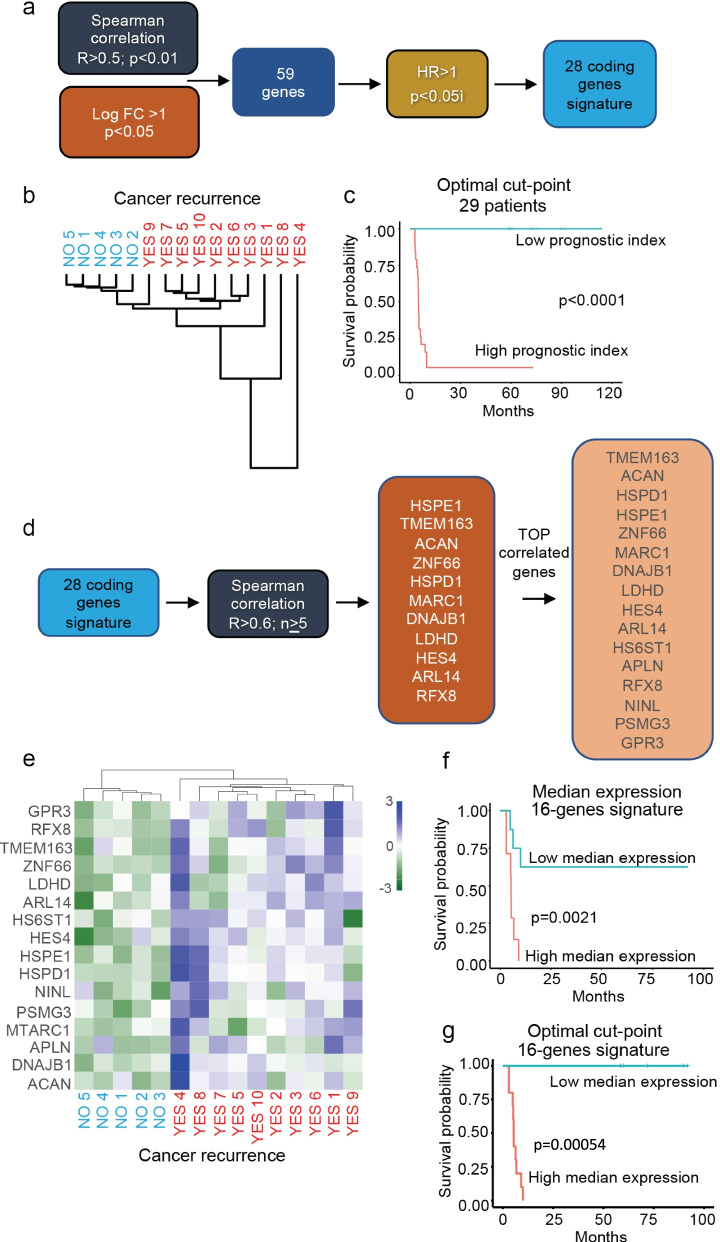
Identification, characterization and prognostic value of the 28-genes risk signature. **a** Signature characterization workflow. Genes were identified based on i) the existence of a high Spearman correlation (R > 0.5, *p*-value< 0.05; *n* = 451) between gene expression and recurrence/progression in the “basic + re-TUR” group of patients, and ii) their up-regulation (logFC> 1.0, p-value< 0.05; *n* = 251) in the “< 1 year recurrence/progression” versus the “> 5 years free status”. Next, a univariate Cox regression analysis with two different significance thresholds identified the subsets of up-regulated genes with HR > 1.0. (R > 0.5, p-value< 0.05; logFC> 1.0; p-value< 0.05; HR > 1.0, p-value< 0.05). **b** Unsupervised hierarchical clustering of patients based on the 28-genes risk signature. Dendrogram of patients clustering, using the Euclidean distance and average linkage methods. **c** Evaluation of the classifying potential of the 28-genes risk signature. Kaplan-Meier plot and a log-rank test were used to determine the statistical significance of the differences in the DFS status of patients stratified using the optimal cut-point of the Prognostic Index. **d** The Spearman correlation of gene expression levels was calculated for all the gene couples in the 28-genes risk signature, identifying all the genes (*n* = 11) with more than five high correlations (R > 0.60) and the top16 gene signature with the highest number of high correlations. **e** Unsupervised hierarchical clustering of patients based on the top16 most-correlated genes. **f** Patients were stratified using the median expression of the top16 most-correlated genes signature. **g** Patients were stratified using the optimal cut-point of the top16 most-correlated genes signature. Kaplan-Meier plot and a log-rank test highlight the statistical significance of the differences in the DFS status

To identify the best classifying genes of the examined patients, we finally focused on those with the most similar expression patterns, calculating the Spearman correlations between all the member of the 28-genes signature. Genes with more than five high correlations (R > 0.60) were identified (*n* = 11 genes) and, for each one, we listed the top-correlated partners, finally picking the top16 most frequently represented genes (Fig. 1d). When tested in unsupervised hierarchical clustering, this signature was the best-performing among all the examined ones, clearly separating patients experiencing recurrence/progression (“yes”, right cluster) from those maintaining a free status (Fig. 1e; “no”, left cluster). This signature also maintained a very good prognostic power: the univariate log-rank test, returned significant results using the median expression (*p*-value = 2.1e-03) (Fig. 1f). This result was further improved when using the optimal cut-point (*p*-value = 5.4e-04) (Fig. 1g).

Gene signatures marking the progression of NMIBC (occurring in about 15% of patients) have long been sought and could constitute the starting point for developing clinical tests predicting whether this circumstance is likely to occur [1, 2]. Following different strategies, we finally identified a signature of 16 genes that has very good prognostic value and clearly separates patients experiencing recurrence/progression. Indeed, these 16 genes are upregulated (although not homogeneously) in patients who relapse/progress, whereas they are often downregulated in patients who do not evidence recurrence/progression for 5 years. Importantly, many of them are never upregulated in patients who do not recur/progress, which is a key criterion for a useful biomarker. A heterogeneous pattern of expression among patients characterizes all of these genes and, in general, the different signatures tested in this study. This observation may partially explain the difficulties encountered in previous attempts at identifying valid biomarkers predicting NMIBC evolution. Among their associated “GO: Biological processes”, these 16 genes control metabolism (*LDHD, MTARC1*), signaling events (*GPR3; ARL14, APLN, TMEM163*), chromosome structure (*NINL*), extracellular matrix (*HS6ST1, ACAN*), protein folding (*DNAJB1, HSPD1, HSPE1*) and transcription (*RFX8, HES4, ZNF66*). Interestingly, in some cases the expression patterns of genes associated with the same pathways show complementary trends among different patients, as in the case of the “protein folding” or the “extracellular matrix” categories (Fig. 1e). This observation suggests that cancer progression may hijack a particular function through alternative strategies.

Focusing only on the *HS6ST1*, *ARL14*, and *RFX8* genes, we found that all patients who experienced recurrence had at least one of these genes upregulated, whereas patients who did not experience recurrence had their expression unchanged or downregulated. These three genes, which have never been associated with NMIBC, may represent the minimal signature required for NMIBC recurrence/progression. RFX8 is a member of a family of transcription factors that bind DNA and that have pleiotropic functions influencing cell cycle, DNA repair and the immune response [3]. *ARL14/ARF7* (ADP ribosylation factor like GTPase 14) encodes a GTPase involved in the regulation of vesicular transport, which was already proposed as a prognostic marker in lung adenocarcinoma. *ARL14* silencing upregulates p16, p27 and p53 expression and tumor dormancy [4]. The third gene, *HS6ST1*, belongs to the heparan sulfate 6-O-sulfotransferase family of enzymes. *HS6ST1* has also been implicated in cancer sustainment, through the regulation of an angiogenic program [5].

In summary, our approach provides a starting point for investigating the presence of these signature genes in biological fluids (i.e. blood/urine) routinely tested in clinical trials, using a larger patient population.

## Supplementary Information

**Additional file 1: Supplementary Table 1.** Clinical-pathological characteristics of the analyzed cohorts. **Supplementary Table 2.** Clinical-pathological characteristics of the GSE154261 dataset. **Supplementary Fig. 1.** Characterization of a 72-genes signature. **Supplementary Fig. 2.** Characterization of a 26-genes signature. **Supplementary Fig. 3.** Characterization of a 16-genes signature.

## Data Availability

The datasets analyzed during the study are available upon reasonable request.

## Abbreviations

NMIBC: Non-muscle-invasive bladder cancer
BCG: Bacillus Calmette-Guérin
TUR: Transurethral resection
DEGs: Differentially expressed genes
HR: Hazard Ratio
DFS: Disease Free State
