# Supplementary material for: Identification of a gene signature for the prediction of recurrence and progression in non-muscle-invasive bladder cancer
Source: Mol Biomed. 2022 Mar 15;3:9. doi: 10.1186/s43556-022-00069-0 (PMC8921352; doi:10.1186/s43556-022-00069-0)
Supplement: Supplementary file 1 — Additional file 1: Supplementary Table 1. Clinical-pathological characteristics of the analyzed cohorts. Supplementary Table 2. Clinical-pathological characteristics of the GSE154261 dataset. Supplementary Fig. 1. Characterization of a 72-genes signature. Supplementary Fig. 2. Characterization of a 26-genes signature. Supplementary Fig. 3. Characterization of a 16-genes signature. [file 43556_2022_69_MOESM1_ESM.pdf]

Supplementary data

Identification of a gene signature for the prediction of recurrence and progression in non-muscle-invasive bladder cancer

Emiliano Dalla, Raffaella Picco, Giacomo Novara, Fabrizio Dal Moro, Claudio Brancolini

MATERIALS AND METHODS

Public Data Processing

RNAseq and clinical data were downloaded from the NCBI GEO repository (https://www.ncbi.nlm.nih.gov/geo/, datasets GSE13507 and GSE154261), from the ArrayExpress archive (https://www.ebi.ac.uk/arrayexpress/, dataset E-MTAB-4321) and from cBioPortal/FireBrowse (https://www.cbioportal.org/ and http://firebrowse.org/, dataset TCGA-BLCA [1-4]).

Patient cohorts and clinical data

Patients from dataset GSE154261 were all newly diagnosed, with no prior bladder cancer, intravesical chemotherapy, or immunotherapy exposure. All patients completed at least induction (6 doses) of full-strength BCG. Recurrence was defined as the development of any high-grade bladder tumor after the last TUR. Any T2 or higher-grade lesion (including metastatic or nodal metastasis) was considered Progression. Time to recurrence was determined as the time from the last TUR procedure to the diagnosis of pathologic recurrence [2].

Bioinformatics Analyses

Differentially expressed genes (DEGs) were identified using the R software package DESeq2 with default parameter settings [5].

Identification of the predictive signatures

A univariate Cox proportional hazard regression model was used [6] to evaluate the association of gene expression levels with the Disease-Free State (DFS). Hazard ratios (HR) from this analysis were used to identify candidate genes significantly ($P < 0.01$) associated with recurrence/progression: genes with $HR < 1$ were considered protective genes, while those with $HR > 1$ were defined as risky genes. For each patient, a Prognostic Index (PI) was calculated as the linear combination of the coefficient (β) derived from the Cox regression analysis multiplied by the gene expression level. Patients were ranked by their PI and divided into two equally sized cohorts, using the median PI as a cutoff value. Kaplan-Meier plots were constructed [7] and a univariate log-rank test was applied to determine differences in DFS of patients with high and low PI.

To evaluate the predictive performance of the proposed gene signatures, we employed time-dependent Receiver Operating Characteristic (ROC) analysis for censored data and area under the curve (AUC) as our criteria to assess recurrence/progression predictions using the R package pROC [8].

The potential of the identified gene lists to classify patients was evaluated based on gene expression and sample clustering. Unsupervised hierarchical clustering analysis was performed in R using the 'hclust' function with Euclidean distance and complete or average linkage methods.

Studies		blca_tcga	E-MTAB-4321	E-GEOD-13507	GSE154261
Cancer types		Bladder Urothelial Carcinoma	Non-Muscle Invasive Bladder Cancer	Non-Muscle Invasive Bladder Cancer	Non-Muscle Invasive Bladder Cancer
Treatment	BCG	37	88	56	73
	no BGC	412	388	47	26
Gender	Female	6	18	12	N.A.
	Male	31	70	44	N.A.
Stage	Ta	0	50	7	0
	T1	0	36	49	73
	CIS	0	2	0	0
	T2	4	0	0	0
	T2a	2	0	0	0
	T2b	2	0	0	0
	T3	5	0	0	0
	T3a	4	0	0	0
	T3b	8	0	0	0
	T4	3	0	0	0
	T4a	5	0	0	0
	T4b	1	0	0	0
	NA	3	0	0	0
DFS	Recur./Progres.	20	4	8	31
	Disease free	15	84	48	42
	N.A,	2	0	0	0

Table 1. Clinical-pathological characteristics of the analyzed cohorts.
The most relevant data, including stage, progression and administered therapy, is shown for each of the four examined datasets, profiled using either expression beadchips (GEO GSE13507) [3] or RNAseq (ArrayExpress E-MTAB-4321) [1], TCGA-BLCA [4] and GEO GSE154261 [2]. We were particularly interested in early stages of tumor development, which led to discarding the TCGA-BLCA dataset (made of treated, advanced studies) as well as datasets GEO GSE13507 and E-MTAB-4321, showing almost no progressing patients. Based on these premises, we selected dataset GSE154261 for further analysis.

Variables	GSE154261
Age - yr (mean)	N.A.
Gender - no. of patients (%)	N.A.
Grade - no. of patients (%)	
High	73 (100)
Stage - no. of patients (%)	
T1	73 (100)
Recurrence - no. of patients (%)	
No	42 (57.5)
Yes	31 (42.5)
Progression - no. of patients (%)	
No	62 (84.9)
Yes	9 (12.3)
NA	2 (2.7)
BCG (Induction)	73 (100)
BCG (Mantenance)	
No	25 (34.2)
Yes	47 (64.4)
N.A.	1 (1.4)
TUR	73 (100)
ReTUR	
No	12 (16.4)
Yes	61 (83.6)

Table 2. Clinical-pathological characteristics of the GSE154261 dataset.
The detailed data, including grade, stage and administration of BCG and/or Re-TUR, is shown for patients of the GSE154261 dataset [2].

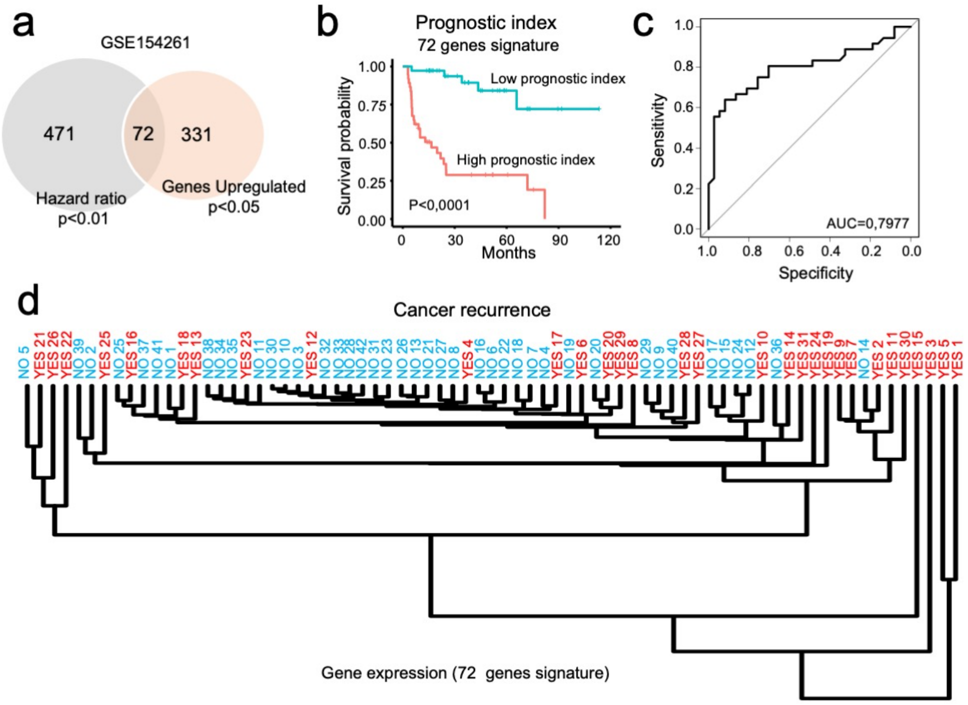

Figure 1. Characterization of a 72-genes signature

a) Definition of the 72-genes signature. Among all the profiled genes (n=58735), we performed a univariate Cox proportional hazard regression analysis to evaluate their Hazard Ratio (HR, the association between their expression levels and the Disease-Free State (DFS)), identifying those associated with an increased risk of disease progression (HR > 1, n=543). In parallel, genes upregulated in patients that experienced cancer recurrence/progression after treatment were selected (n=403) (abs(logFC)>1, p-value≤0.05). The risky genes that were also upregulated constituted the first gene signature (n=72) that we tested.

b) For each patient we calculated the Prognostic Index, using it to rank patients and dividing them into two equally sized cohorts, using the median Index value as a cutoff. We applied a univariate log-rank test to determine the difference in DFS for patients with high and low Prognostic Index representing the outcome using a Kaplan-Meier plot, clearly demonstrating the predictive potential of this gene signature.

c) To further evaluate the 72-genes signature predictive performance, we employed time-dependent Receiver Operating Characteristic (ROC) analysis for censored data and area under the curve (AUC) as our criteria to assess recurrence/progression predictions.

d) Despite having obtained significant results in the survival analysis, the identified gene signature was not able to perfectly classify the two groups of patients when performing unsupervised hierarchical clustering.

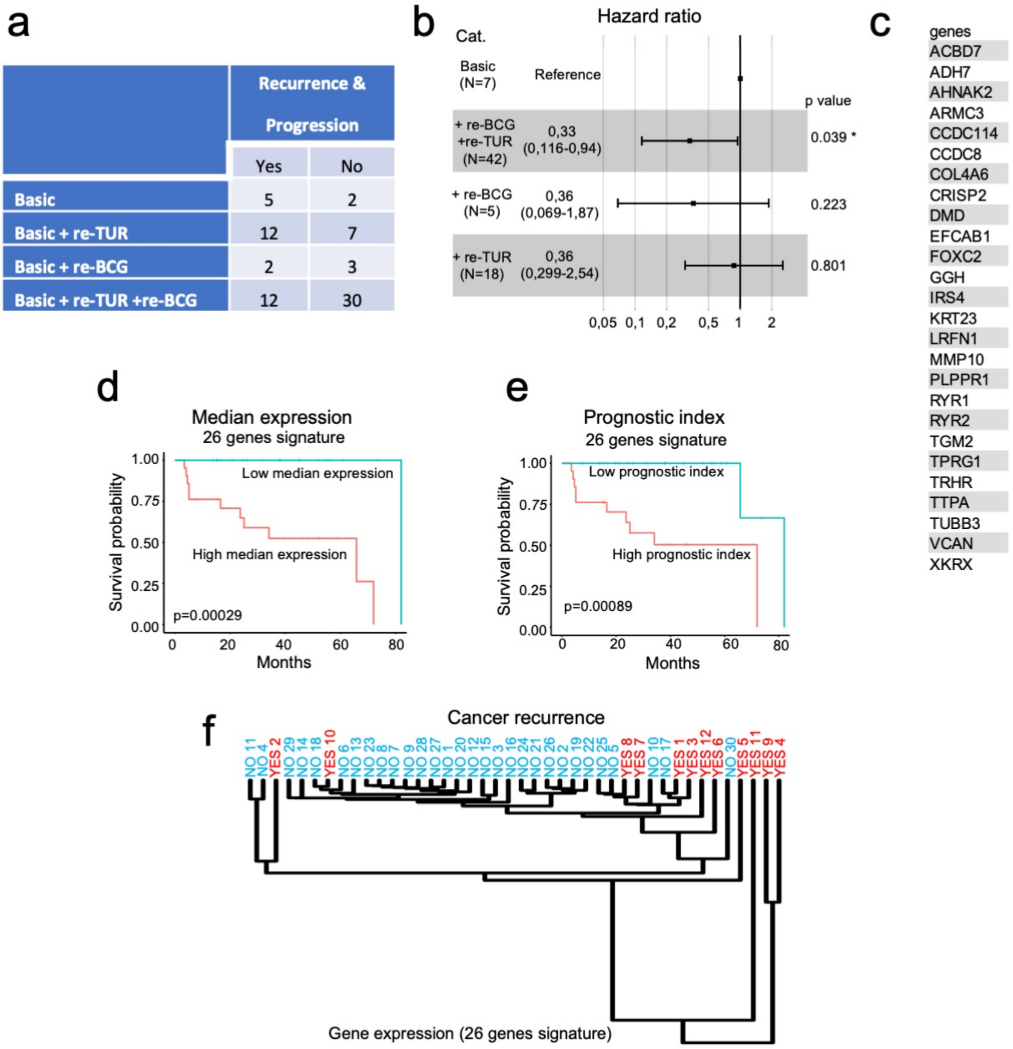

Figure 2. Characterization of a 26-genes signature

The failure of the first approach prompted us to follow a different strategy.

a) Patients were classified into four groups based on the type of treatment received: Induction administration (6 doses) of full-strength BCG + TUR (basic); basic + repeated TUR (basic + re-TUR); basic + maintenance BCG treatment (basic + re-BCG); basic + maintenance BCG treatment + repeated TUR (basic + re-TUR + re-BCG) [2].

b) The previous analysis (Supplementary Fig. 1) was repeated. DEGs and their HR for each of the four examined groups were defined. Next, we performed a multivariate Cox proportional hazard regression analysis finding that only group "basic + re-TUR + re-BCG" could be used as an independent predictor.

c) List of protein-coding genes that are upregulated in patients' group "basic + re-TUR + re-BCG" and that are associated with an increased risk of disease progression (HR > 1, n=26).

d) Survival probability of patients stratified using the 26-genes risk signature. Patients are stratified using the median expression.

e) Survival probability of patients stratified using the 26-genes risk signature. Patients are stratified using median Prognostic Index. Kaplan-Meier plots and a log-rank test are used to determine the statistical significance of the differences in the DFS status.

f) Unsupervised hierarchical clustering of patients based on the 26-genes risk signature. Dendrogram of patients clustering, using the Euclidean distance and average linkage methods.

This figure demonstrates the predictive potential of this second 26-gene signature. However, also this signature failed in classifying the two groups of patients when performing unsupervised hierarchical clustering [Fig. 1H], probably due to the impact of the heterogeneity of the follow-up time on the definition of the patient status. Some patients, in fact, were monitored for a sufficiently long time (n=12 patients > 60 months (max. 113 months)), allowing to properly define the recurrence state, while others were under examination for a shorter time (n=13 patients < 6 months (min. 3 months)) and the attributed patient status could not be definitive, with the reappearance that might only be postponed.

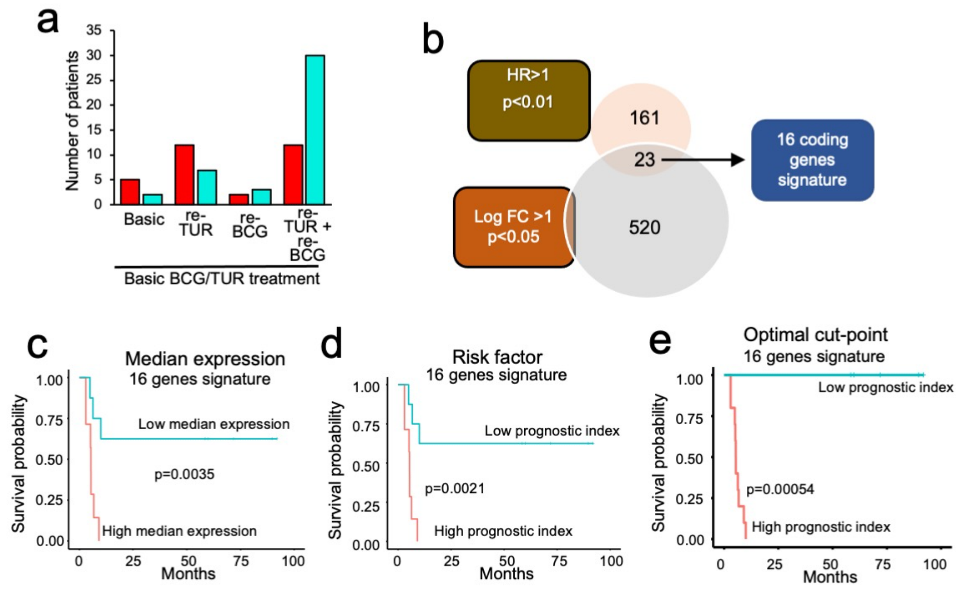

Figure 3. Characterization of a 16-genes signature

a) Application of the third strategy. Within each type of treatment, we compared patients experiencing an early recurrence/progression within 12 months to patients that were still free from recurrence/progression after a period of 5 years or more. Patients are classified into four groups based on the type of treatment received: induction administration (6 doses) of full-strength BCG + TUR (basic); basic + repeated TUR (basic + re-TUR); basic + maintenance BCG treatment (basic + re-BCG); basic + maintenance BCG treatment + repeated TUR (basic + re-TUR + re-BCG). In this way, we identified group “basic + re-TUR” as the one associated with the highest number of patients with a < 1 year recurrence/progression (n=10) and with a > 5 years free status (n=5).

b) We decided to focus on the “basic + re-TUR” group as it seemed the most promising for identifying the genes related to the effectiveness of the treatment and those responsible for its refractoriness. Patients belonging to the “basic + re-TUR” group were profiled identifying i) genes associated with an increased risk of disease progression (HR > 1, p < 0.01; n=161) and ii) genes that were up-regulated (logFC>1.0, p < 0.01; n=520) in patients undergoing recurrence/progression with respect to patients with a free status. 16 protein-coding genes were identified.

c) Demonstration of the predictive potential of the 16-genes signature. Patients stratification using the median expression of the top16 most-correlated genes signature (p-value=3.5e-03).

d) Demonstration of the predictive potential of the 16-genes signature. Patients stratification using the median prognostic Index of the top16 most-correlated genes signature (p-value=2.1e-03).

e) Demonstration of the predictive potential of the 16-genes signature. Patients stratification using the optimal cut-point prognostic Index of the top16 most-correlated genes signature (p-value=5.4e-04).

Interestingly, this unbiased approach perfectly distinguished patients with < 1 year recurrence/progression from those with > 5 years free status. Unfortunately, this signature did not perform better than the previous ones in terms of grouping patients upon unsupervised hierarchical clustering (data not shown).

REFERENCES

1. Hedegaard J, Lamy P, Nordentoft I, Algaba F, Høyer S, Ulhøi BP, et al. Comprehensive transcriptional analysis of early-stage urothelial carcinoma. Cancer Cell. 2016 Jul 11;30(1):27-42. doi: 10.1016/j.ccell.2016.05.004.

2. Robertson AG, Groeneveld CS, Jordan B, Lin X, McLaughlin KA, Das A, et al. Identification of differential tumor subtypes of T1 bladder cancer. Eur Urol. 2020 Oct;78(4):533-537. doi: 10.1016/j.eururo.2020.06.048.

3. Kim WJ, Kim EJ, Kim SK, Kim YJ, Ha YS, Jeong P, et al. Predictive value of progression-related gene classifier in primary non-muscle invasive bladder cancer. Mol Cancer. 2010 Jan 8;9:3. doi: 10.1186/1476-4598-9-3.

4. Robertson AG, Kim J, Al-Ahmadie H, Bellmunt J, Guo G, Cherniack AD, et al. Comprehensive molecular characterization of muscle-invasive bladder cancer. Cell. 2017;171(3):540-556.e25. doi: 10.1016/j.cell.2017.09.007.

5. Love MI, Huber W, Anders S. Moderated estimation of fold change and dispersion for RNA-seq data with DESeq2. Genome Biol. 2014;15(12):550. doi: 10.1186/s13059-014-0550-8.

6. Therneau T (2021). A package for survival analysis in R. R package version 3.2-13, https://CRAN.R-project.org/package=survival

7. Kassambara A, Kosinski M, Biecek P (2021). Survminer: drawing survival curves using 'ggplot2'. R package version 0.4.9. https://CRAN.R-project.org/package=survminer

8. Robin X, Turck N, Hainard A, Tiberti N, Lisacek F, Sanchez JC, et al. pROC: an open-source package for R and S+ to analyze and compare ROC curves. BMC Bioinformatics. 2011 Mar 17;12:77. doi: 10.1186/1471-2105-12-77.
